# Rates of Influenza-Associated Hospitalization, Intensive Care Unit Admission, and In-Hospital Death by Race and Ethnicity in the United States From 2009 to 2019

**DOI:** 10.1001/jamanetworkopen.2021.21880

**Published:** 2021-08-24

**Authors:** Alissa C. O’Halloran, Rachel Holstein, Charisse Cummings, Pam Daily Kirley, Nisha B. Alden, Kimberly Yousey-Hindes, Evan J. Anderson, Patricia Ryan, Sue Kim, Ruth Lynfield, Chelsea McMullen, Nancy M. Bennett, Nancy Spina, Laurie M. Billing, Melissa Sutton, William Schaffner, H. Keipp Talbot, Andrea Price, Alicia M. Fry, Carrie Reed, Shikha Garg

**Affiliations:** 1 Influenza Division, National Center for Immunization and Respiratory Diseases, Centers for Disease Control and Prevention, Atlanta, Georgia; 2 Abt Associates, Rockville, Maryland; 3 California Emerging Infections Program, Oakland; 4 Communicable Disease Branch, Colorado Department of Public Health and Environment, Denver; 5 Connecticut Emerging Infections Program, Yale School of Public Health, New Haven; 6 Departments of Medicine and Pediatrics, Emory University School of Medicine, Atlanta, Georgia; 7 Emerging Infections Program, Georgia Department of Health, Atlanta; 8 Veterans Affairs Medical Center, Atlanta, Georgia; 9 Maryland Department of Health, Baltimore; 10 Communicable Disease Division, Michigan Department of Health and Human Services, Lansing; 11 Minnesota Department of Health, St. Paul; 12 New Mexico Department of Health, Santa Fe; 13 University of Rochester School of Medicine and Dentistry, Rochester, New York; 14 New York State Department of Health, Albany; 15 Bureau of Infectious Diseases, Ohio Department of Health, Columbus; 16 Public Health Division, Oregon Health Authority, Portland; 17 Division of Infectious Diseases, Vanderbilt University School of Medicine, Nashville, Tennessee; 18 Salt Lake County Health Department, Salt Lake City, Utah

## Abstract

**Importance:**

Racial and ethnic minority groups, such as Black, Hispanic, American Indian or Alaska Native, and Asian or Pacific Islander persons, often experience higher rates of severe influenza disease.

**Objective:**

To describe rates of influenza-associated hospitalization, intensive care unit (ICU) admission, and in-hospital death by race and ethnicity over 10 influenza seasons.

**Design, Setting, and Participants:**

This cross-sectional study used data from the Influenza-Associated Hospitalization Surveillance Network (FluSurv-NET), which conducts population-based surveillance for laboratory-confirmed influenza-associated hospitalizations in selected counties, representing approximately 9% of the US population. Influenza hospitalizations from the 2009 to 2010 season to the 2018 to 2019 season were analyzed. Data were analyzed from October 2020 to July 2021.

**Main Outcomes and Measures:**

The main outcomes were age-adjusted and age-stratified rates of influenza-associated hospitalization, ICU admission, and in-hospital death by race and ethnicity overall and by influenza season.

**Results:**

Among 113 352 persons with an influenza-associated hospitalization (34 436 persons [32.0%] aged ≥75 years; 61 009 [53.8%] women), 70 225 persons (62.3%) were non-Hispanic White (White), 24 850 persons (21.6%) were non-Hispanic Black (Black), 11 903 persons (10.3%) were Hispanic, 5517 persons (5.1%) were non-Hispanic Asian or Pacific Islander, and 857 persons (0.7%) were non-Hispanic American Indian or Alaska Native. Among persons aged younger than 75 years and compared with White persons of the same ages, Black persons were more likely to be hospitalized (eg, age 50-64 years: rate ratio [RR], 2.50 95% CI, 2.43-2.57) and to be admitted to an ICU (eg, age 50-64 years: RR, 2.09; 95% CI, 1.96-2.23). Among persons aged younger than 50 years and compared with White persons of the same ages, American Indian or Alaska Native persons were more likely to be hospitalized (eg, age 18-49 years: RR, 1.72; 95% CI, 1.51-1.96) and to be admitted to an ICU (eg, age 18-49 years: RR, 1.84; 95% CI, 1.40-2.42). Among children aged 4 years or younger and compared with White children, hospitalization rates were higher in Black children (RR, 2.21; 95% CI, 2.10-2.33), Hispanic children (RR, 1.87; 95% CI, 1.77-1.97), American Indian or Alaska Native children (RR, 3.00; 95% CI, 2.55-3.53), and Asian or Pacific Islander children (RR, 1.26; 95% CI, 1.16-1.38), as were rates of ICU admission (Black children: RR, 2.74; 95% CI, 2.43-3.09; Hispanic children: RR, 1.96; 95% CI, 1.73-2.23; American Indian and Alaska Native children: RR, 3.51; 95% CI, 2.45-5.05). In this age group and compared with White children, in-hospital death rates were higher among Hispanic children (RR, 2.98; 95% CI, 1.23-7.19), Black children (RR, 3.39; 95% CI, 1.40-8.18), and Asian or Pacific Islander children (RR, 4.35; 95% CI, 1.55-12.22). Few differences were observed in rates of severe influenza-associated outcomes by race and ethnicity among adults aged 75 years or older. For example, in this age group, compared with White adults, hospitalization rates were slightly higher only among Black adults (RR, 1.05; 95% CI 1.02-1.09). Overall, Black persons had the highest age-adjusted hospitalization rate (68.8 [95% CI, 68.0-69.7] hospitalizations per 100 000 population) and ICU admission rate (11.6 [95% CI, 11.2-11.9] admissions per 100 000 population).

**Conclusions and Relevance:**

This cross-sectional study found racial and ethnic disparities in rates of severe influenza-associated disease. These data identified subgroups for whom improvements in influenza prevention efforts could be targeted.

## Key Points

**Question:**

Are rates of severe influenza disease associated with race and ethnicity?

**Findings:**

In this cross-sectional study of influenza-associated outcomes among 113 352 patients hospitalized with influenza over the course of 10 influenza seasons, Black, Hispanic, and American Indian or Alaska Natives persons had higher rates of hospitalization and intensive care unit admission, even after adjusting for age. The greatest disparities were found in the youngest age groups.

**Meaning:**

These findings suggest that targeted prevention and intervention efforts, such as improved influenza vaccine coverage and early use of antiviral treatment, could improve influenza-associated outcomes among racial and ethnic minority groups identified in this study as having higher rates of severe influenza disease.

## Teaser

This cross-sectional study examines rates of influenza-associated hospitalization, intensive care unit (ICU) admission, and in-hospital death by race and ethnicity in the US from 2009 to 2019.

## Introduction

The burden of influenza in the United States is substantial, and annual influenza epidemics result in 140 000 to 810 000 hospitalizations and 12 000 to 61 000 deaths each year.^1^ The ongoing COVID-19 pandemic has highlighted striking racial and ethnic disparities in COVID-19–associated morbidity and mortality^2,3,4,5,6,7^ and raised important questions about racial and ethnic disparities associated with other respiratory viral infections, including influenza. Prior research has shown that owing, in part, to limitations of public health infrastructure and disparities in health care, pandemic influenza outbreaks are likely to disproportionately affect socially disadvantaged persons in the United States.^8^ During the 2009 influenza A H1N1 pandemic, racial and ethnic disparities in influenza-associated hospitalizations and pediatric deaths were identified,^9^ and non-Hispanic American Indian or Alaska Native persons were found to have 3- to 8-fold higher hospitalization and death rates associated with influenza A(H1N1)pdm09 compared with other racial and ethnic groups.^10^ Since that time, non-Hispanic American Indian or Alaska Native persons have been considered by the Advisory Committee on Immunization Practices to be at increased risk for influenza-associated complications.^11^ Disparities in seasonal influenza-associated hospitalizations have also been observed^12^; non-Hispanic Black persons have been reported to have 1.7-fold higher adjusted odds of hospitalization, and Hispanic or Latino persons have been reported to have 1.2-fold higher adjusted odds of hospitalization compared with non-Hispanic White persons in a study conducted during the 2009 to 2010 through 2013 to 2014 influenza seasons.^13^ However, recent comprehensive data on racial and ethnic differences in rates of influenza-associated hospitalizations in the United States are lacking. We analyzed data from the Centers for Disease Control and Prevention (CDC)’s Influenza-Associated Hospitalization Surveillance Network (FluSurv-NET) to estimate rates of influenza-associated hospitalization, intensive care unit (ICU) admission, and in-hospital death by race and ethnicity over 10 influenza seasons (2009-2010 through 2018-2019).

## Methods

FluSurv-NET surveillance activities were reviewed by CDC and were conducted consistent with applicable federal law and CDC policy (eg, 45 CFR. part 46.102[l][2], 21 CFR part 56; 42 USC §241[d]; 5 USC §552a; 44 USC §3501 et seq.). Sites participating in FluSurv-NET obtained approval from their respective state and local institutional review boards, as applicable. The requirement for informed consent was waived per 45 CFR 46. This study is reported following the Strengthening the Reporting of Observational Studies in Epidemiology (STROBE) reporting guideline.

### Description of FluSurv-NET Surveillance

FluSurv-NET, which has been previously described,^14^ conducts all-age population-based surveillance for laboratory-confirmed influenza-associated hospitalizations in selected counties in states that participate in the Emerging Infections Program and Influenza Hospitalization Surveillance Project (eFigure 1 in the Supplement). Emerging Infections Program sites include selected counties in California, Colorado, Connecticut, Georgia, Maryland, Minnesota, New Mexico, New York, Oregon, and Tennessee, while Influenza Hospitalization Surveillance Project sites include selected counties in Idaho (2009-2010 to 2010-2011 only), Iowa (2009-2010 and 2012-2013 only), Michigan, North Dakota (2009-2010 only), Ohio (2010-2011 to 2018-2019), Oklahoma (2009-2010 to 2010-2011 only), Rhode Island (2010-2011 to 2012-2013 only), South Dakota (2009-2010 only) and Utah (2010-2011 to 2018-2019). Approximately 25 to 29 million persons (8%-9% of the US population) were in the catchment of this surveillance system across these seasons.

Persons met the FluSurv-NET inclusion criteria if they were a resident of the FluSurv-NET catchment area, had a hospital admission date between October 1 and April 30 (or April 15, 2009, through April 30, 2010, during the 2009 influenza A H1N1 pandemic season), and had a positive influenza test no more than 14 days prior to or during hospitalization. Influenza testing was ordered at the discretion of health care practitioners, and laboratory confirmation was defined by a positive test result from rapid antigen diagnostic testing, molecular assay, indirect or direct fluorescent antibody assay, or viral culture. Trained surveillance staff conducted medical record abstractions using a standardized data collection form to obtain information on demographics, including age, sex, race and ethnicity, underlying medical conditions, antiviral treatment, and outcomes, including ICU admission and in-hospital death.

Race and ethnicity data were obtained from multiple sources, including notifiable disease, laboratory, and hospital databases. In most cases, race and ethnicity is self-reported, but the source could not be confirmed in every case. We categorized race and ethnicity according to the National Center for Health Statistics categories as non-Hispanic White, non-Hispanic Black, Hispanic or Latino, non-Hispanic Asian or Pacific Islander, and non-Hispanic American Indian or Alaska Native. People of more than 1 race and ethnicity or unknown race and ethnicity are captured by surveillance but were excluded from this analysis, since population denominators were not available for these groups. People of any race (including unknown race) but of Hispanic ethnicity were classified as Hispanic. If ethnicity was unknown (16.9% of included persons) but race was available, persons were classified based on reported race but were assumed to be of non-Hispanic ethnicity.

### FluSurv-NET Sampling and Weighting Methodology

A minimum data set (including age, surveillance site, hospital admission date, and positive influenza test result and date) was reported for all persons across all seasons. During the 2009 to 2010 through 2016 to 2017 influenza seasons, medical record abstractions were also performed for all persons. Because of high counts of laboratory-confirmed influenza-associated hospitalizations during the 2017 to 2018 and 2018 to 2019 seasons, an age- and site-stratified random sampling scheme was used to obtain detailed clinical data through medical record abstraction for a representative sample of adults aged 50 years or older in 2017 to 2018 and adults aged 65 years or older in 2018 to 2019, as previously described.^15^ Medical record abstractions were conducted for all patients younger than 50 years and all patients of any age who died during their hospitalization. Of 14 surveillance sites included during these 2 seasons, 7 opted to implement a sampling strategy during the 2017 to 2018 season and 6 used the sampling strategy during the 2018 to 2019 season (Table 1). Race and ethnicity (2017-2018) and clinical data (2017-2018 and 2018-2019) on underlying conditions, receipt of at least 1 dose of current season influenza vaccine (for patients aged ≥6 months), ICU admission status, and antiviral treatment were only collected for sampled persons.

**Table 1.** Demographic Characteristics of Persons With Laboratory-Confirmed Influenza-Associated Hospitalizations by Race and Ethnicity

Characteristic	Unweighted No. (weighted %)					
	Total (N = 113 352)	Non-Hispanic				Hispanic (n = 11 903)
		White (n = 70 225)	Black (n = 24 850)	American Indian or Alaska Native (n = 857)	Asian or Pacific Islander (n = 5117)	
Age, y						
≤4	8697 (7.2)	3097 (4.1)	2472 (9.5)	154 (17.3)	599 (9.6)	2375 (19.1)
5-17	6417 (5.3)	2787 (3.7)	1951 (7.5)	64 (7.2)	330 (5.3)	1285 (10.3)
18-49	21 632 (17.9)	10 147 (13.4)	7027 (26.9)	235 (26.4)	948 (15.2)	3275 (26.4)
50-64	24 321 (21.2)	14 408 (20.0)	6847 (27.8)	211 (24.3)	837 (14.5)	2018 (17.4)
65-74	17 849 (16.5)	12 477 (18.4)	3233 (13.9)	85 (11.1)	824 (15.9)	1230 (10.8)
≥75	34 436 (32.0)	27 309 (40.4)	3320 (14.4)	108 (13.8)	1979 (39.5)	1720 (16.0)
Sex						
Men	52 343 (46.2)	32 822 (46.7)	10 801 (43.6)	394 (45.9)	2695 (48.4)	5631 (47.1)
Women	61 009 (53.8)	37 403 (53.3)	14 049 (56.4)	463 (54.1)	2822 (51.6)	6272 (52.9)
Site^a^						
California^b^	12 411 (12.4)	5276 (8.8)	2254 (10.0)	48 (5.8)	2664 (51.9)	2169 (19.6)
Colorado	10 431 (8.8)	6749 (9.1)	1103 (4.3)	78 (9.2)	321 (5.2)	2180 (18.0)
Connecticut	7942 (6.7)	5403 (7.3)	1513 (5.9)	10 (1.2)	60 (1.0)	956 (7.9)
Georgia^b^	10 197 (9.7)	3871 (6.2)	5153 (22.3)	17 (2.0)	299 (5.4)	857 (7.6)
Maryland	13 784 (11.6)	7279 (9.9)	5822 (22.6)	24 (2.8)	306 (5.0)	353 (2.9)
Michigan	3344 (2.8)	2418 (3.3)	770 (3.0)	13 (1.5)	36 (0.6)	107 (0.9)
Minnesota^c^	10 881 (10.1)	7636 (11.7)	1960 (7.9)	156 (19.1)	647 (11.2)	482 (4.1)
New Mexico^b^	4409 (4.2)	1748 (2.7)	127 (0.5)	341 (43.5)	47 (0.9)	2146 (19.3)
New York						
Albany^b^	4942 (4.8)	4011 (6.3)	642 (2.7)	4 (0.5)	81 (1.6)	204 (1.8)
Rochester	8869 (7.5)	6513 (8.8)	1575 (6.1)	16 (1.9)	107 (1.7)	658 (5.4)
Ohio^b^	6691 (6.6)	4685 (7.6)	1715 (7.3)	15 (1.9)	121 (2.2)	155 (1.4)
Oregon^b^	5912 (5.6)	4679 (7.2)	407 (1.7)	51 (6.2)	320 (6.0)	455 (3.9)
Tennessee	6297 (5.3)	4606 (6.2)	1382 (5.4)	4 (0.5)	80 (1.3)	225 (1.9)
Utah	4841 (4.1)	3652 (5.0)	120 (0.5)	32 (3.8)	375 (6.1)	662 (5.5)

^a^ Site-level data not shown for 2401 persons (2%) from 6 sites who participated in FluSurv-NET surveillance for 3 or fewer of 10 influenza seasons (Iowa, Idaho, North Dakota, Oklahoma, Rhode Island, South Dakota).

^b^ During the 2017 to 2018 season, California, Georgia, New Mexico, Ohio, Oregon, and Albany, New York, conducted medical record abstractions on a 50% random sample of adults aged 50 to 64 years and a 25% random sample of adults aged 65 years or older. During the 2018 to 2019 season, these same sites conducted medical record abstractions on a 50% random sample of adults aged 65 years or older. These sites conducted medical chart abstractions on 100% of persons in all other age groups and seasons.

^c^ During the 2017 to 2018 season, Minnesota conducted medical record abstractions on a 50% random sample of adults aged 65 years or older. Minnesota conducted medical record abstractions on 100% of persons in all other age groups and seasons.

For all seasons, sampled data were weighted using survey procedures in SAS statistical software version 9.4 (SAS Institute), in which the weight was the inverse probability of selection of a person for medical record abstraction. For seasons prior to 2017 to 2018, sample weights were equal to 1. For the 2017 to 2018 season, sampling rates ranged from 25% to 100% by age group and site, while in 2018 to 2019, sampling rates ranged from 50% to 100% (Table 1), yielding weights ranging from 1 to 4. The weighted distribution of demographic characteristics, underlying medical conditions, influenza vaccination receipt, and antiviral treatment were calculated by age group and race and ethnicity; unweighted sample sizes and weighted percentages are presented.

### Statistical Analysis

Population denominators used for rate estimation were obtained from the National Center for Health Statistics bridged-race population estimates.^16^ These files have postcensal and intercensal estimates of the US resident population for the indicated years, by county, age, bridged race, Hispanic origin, and sex. For the 2010 to 2011 through 2018 to 2019 seasons, Vintage 2010 to 2018 postcensal estimates were applied. For the 2009 to 2010 season, the 2009 intercensal estimate was applied.

Unadjusted rates were calculated by dividing the weighted number of hospitalizations, ICU admissions, or in-hospital deaths by the total catchment population. Unadjusted rates were stratified by age group (0-4, 5-17, 18-49, 50-64, 65-74, and ≥75 years) and race and ethnicity. Unadjusted rates by race and ethnicity and age group were multiplied by the age distribution of the total FluSurv-NET catchment population to obtain age-adjusted rates; the age groups referenced previously were used for the age adjustment. We calculated 95% CIs for rates and rate ratios (RRs) assuming a simple random sample design and a normal distribution via the SAS STDRATE procedure. All analyses were conducted using SAS software. *P* values were 2-sided, and statistical significance was set at α = .05. Data were analyzed from October 2020 to July 2021.

## Results

### Demographic and Clinical Characteristics of Hospitalized Persons by Race and Ethnicity

Across all seasons, 131 908 persons hospitalized with influenza were identified, and of those 123 882 persons (93.9%) were sampled. Among the 123 882 sampled persons, 10 037 (8.1%) were excluded because race and ethnicity were unknown and 493 (0.4%) were excluded because they were classified as having more than 1 race and ethnicity. Table 1 displays the distribution of the remaining 113 352 persons (34 436 persons [32.0%] aged ≥75 years; 61 009 [53.8%] women) included in the analysis across seasons. Overall, the sample included 70 225 White persons (62.3%), 24 850 Black persons (21.6%), 11 903 Hispanic persons (10.3%), 5517 Asian or Pacific Islander persons (5.1%), and 857 American Indian or Alaska Native persons (0.7%). Among hospitalized persons, the prevalence of underlying conditions varied by age and race and ethnicity (eTable 1 in the Supplement). For example, compared with White children aged 17 years or younger, Black children had higher prevalence of asthma and blood disorders. Black persons and Hispanic persons had lower vaccination coverage compared with White persons in all age groups (eTable 1 in the Supplement). Differences in antiviral treatment were noted across age groups but not by race and ethnicity within the same age group (eTable 1 in the Supplement).

### Age-Specific Rates of Influenza-Associated Hospitalization, ICU Admission, and In-Hospital Death by Race and Ethnicity

**Figure 1.**
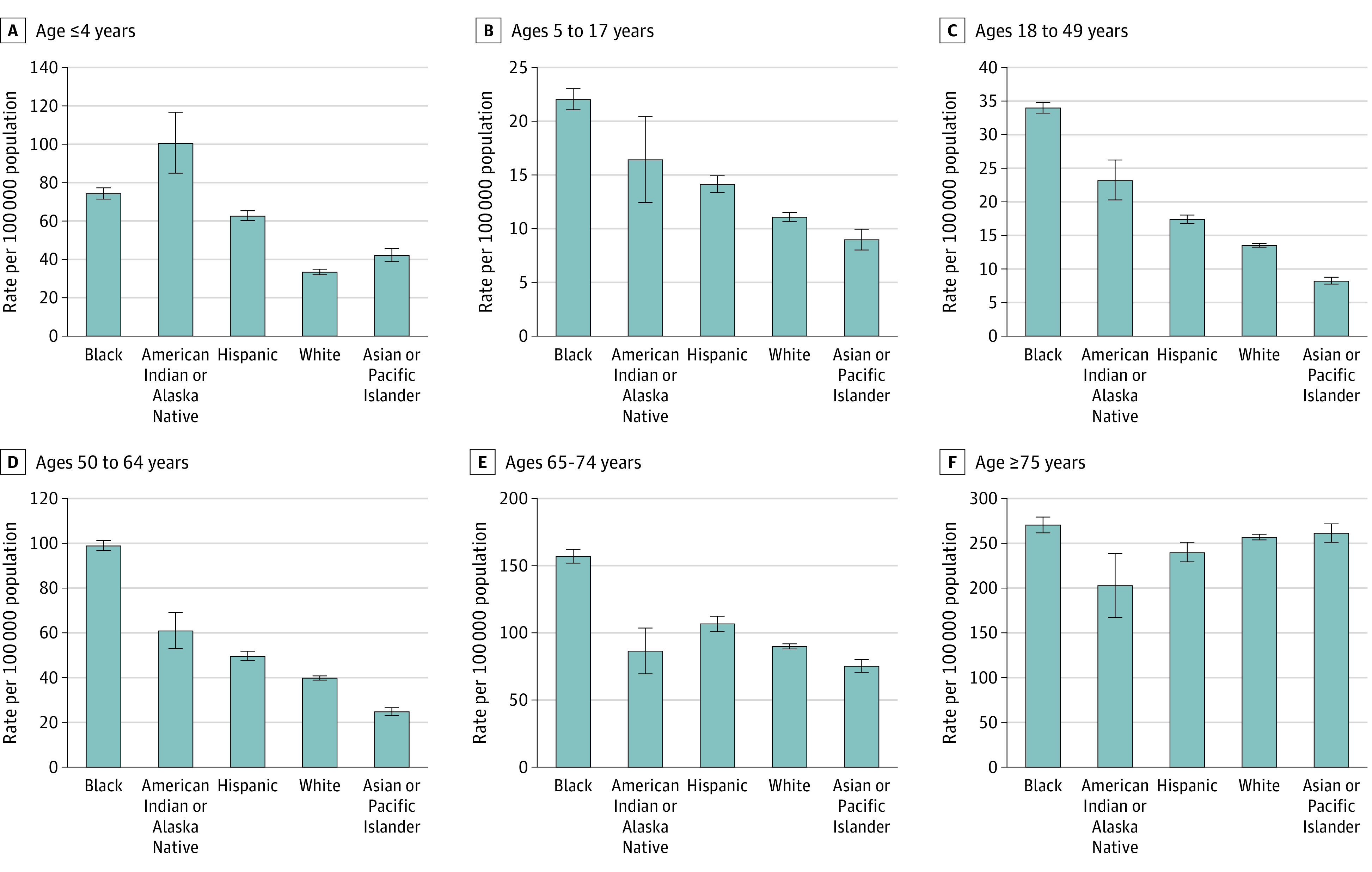
Age-Specific Rates of Hospitalization by Race and Ethnicity

Unadjusted age-specific hospitalization (Figure 1) and ICU admission rates (eTable 2 in the Supplement) were highest among Black persons for all age groups except age 4 years or younger, in which hospitalization and ICU admission rates were highest among American Indian or Alaska Native persons. Compared with White persons, unadjusted hospitalization RRs varied by age group and were higher among Black persons younger than 75 years (eg, age 50-64 years: RR, 2.50, 95% CI, 2.43-2.57), American Indian or Alaska Native persons younger than 65 years (eg, age 18-49 years: RR, 1.72; 95% CI, 1.51-1.96), and Hispanic children aged 4 years or younger (RR, 1.87; 95% CI, 1.77-1.97) (Table 2). Compared with White persons, unadjusted ICU admission RRs were higher among Black persons younger than 75 years (eg, age 50-64 years: RR, 2.09; 95% CI, 1.96-2.23), American Indian or Alaska Native persons younger than 50 years (eg, age 18-49 years: RR, 1.84; 95% CI, 1.40-2.42), and Hispanic children younger than 4 years (RR, 1.96; 95% CI, 1.73-2.23) (Table 2).

**Table 2.** Age-Specific Rate Ratios of Hospitalization, ICU Admission, and In-Hospital Death by Race and Ethnicity

Outcome	Rate ratio (95% CI)				
	Non-Hispanic				Hispanic
	White	Black	American Indian or Alaska Native	Asian or Pacific Islander	
Hospitalization, age group, y					
≤4	1 [Reference]	2.21 (2.10-2.33)	3.00 (2.55-3.53)	1.26 (1.16-1.38)	1.87 (1.77-1.97)
5-17	1 [Reference]	1.99 (1.88-2.11)	1.48 (1.16-1.90)	0.81 (0.72-0.91)	1.28 (1.19-1.36)
18-49	1 [Reference]	2.52 (2.44-2.59)	1.72 (1.51-1.96)	0.61 (0.57-0.65)	1.29 (1.24-1.34)
50-64	1 [Reference]	2.50 (2.43-2.57)	1.54 (1.34-1.76)	0.63 (0.59-0.67)	1.25 (1.20-1.31)
65-74	1 [Reference]	1.74 (1.68-1.81)	0.96 (0.79-1.17)	0.84 (0.78-0.89)	1.18 (1.12-1.25)
≥75	1 [Reference]	1.05 (1.02-1.09)	0.79 (0.66-0.94)	1.02 (0.98-1.06)	0.93 (0.89-0.98)
ICU admission, age group, y					
≤4	1 [Reference]	2.74 (2.43-3.09)	3.51 (2.45-5.05)	1.31 (1.06-1.61)	1.96 (1.73-2.23)
5-17	1 [Reference]	2.00 (1.77-2.26)	1.88 (1.18-3.00)	0.97 (0.78-1.22)	1.16 (1.00-1.34)
18-49	1 [Reference]	1.85 (1.72-1.99)	1.84 (1.40-2.42)	0.57 (0.49-0.66)	1.14 (1.04-1.24)
50-64	1 [Reference]	2.09 (1.96-2.23)	1.17 (0.84-1.63)	0.61 (0.53-0.71)	1.04 (0.93-1.15)
65-74	1 [Reference]	1.50 (1.37-1.64)	1.34 (0.91-1.98)	0.87 (0.75-1.00)	1.11 (0.98-1.27)
≥75	1 [Reference]	1.26 (1.15-1.37)	0.72 (0.42-1.21)	1.21 (1.08-1.34)	0.88 (0.77-1.00)
In-hospital death, age group, y					
≤4	1 [Reference]	3.39 (1.40-8.18)	6.71 (0.85-52.97)	4.35 (1.55-12.22)	2.98 (1.23-7.19)
5-17	1 [Reference]	1.19 (0.62-2.28)	4.17 (1.00-17.41)	1.55 (0.68-3.51)	0.80 (0.38-1.69)
18-49	1 [Reference]	1.22 (0.94-1.57)	2.20 (1.04-4.67)	0.55 (0.35-0.87)	1.07 (0.81-1.41)
50-64	1 [Reference]	1.53 (1.28-1.83)	1.24 (0.55-2.77)	0.46 (0.31-0.70)	1.08 (0.83-1.40)
65-74	1 [Reference]	1.19 (0.94-1.51)	0.60 (0.15-2.42)	1.00 (0.72-1.39)	1.07 (0.77-1.48)
≥75	1 [Reference]	0.93 (0.79-1.10)	0.44 (0.14-1.35)	1.22 (1.02-1.46)	0.71 (0.56-0.91)

Abbreviation: ICU, intensive care unit.

Compared with White persons of the same age group, unadjusted death rates were higher among Black adults aged 50 to 64 years (RR, 1.53; 95% CI, 1.28-1.83), American Indian or Alaska Native adults aged 18 to 49 years (RR, 2.20; 95% CI, 1.04-4.67), and American Indian or Alaska Native children aged 5 to 17 years (RR, 4.17; 95% CI, 1.00-17.41) (Table 2). Among children aged 4 years or younger and compared with White children, unadjusted in-hospital death rates were higher among Hispanic children (RR, 2.98; 95% CI, 1.23-7.19), Black children (RR, 3.39; 95% CI, 1.40-8.18), and Asian or Pacific Islander children (RR, 4.35; 95% CI, 1.55-12.22) (Table 2).

### Age-Adjusted Rates of Hospitalization, ICU Admission, and In-Hospital Mortality by Race and Ethnicity

**Figure 2.**
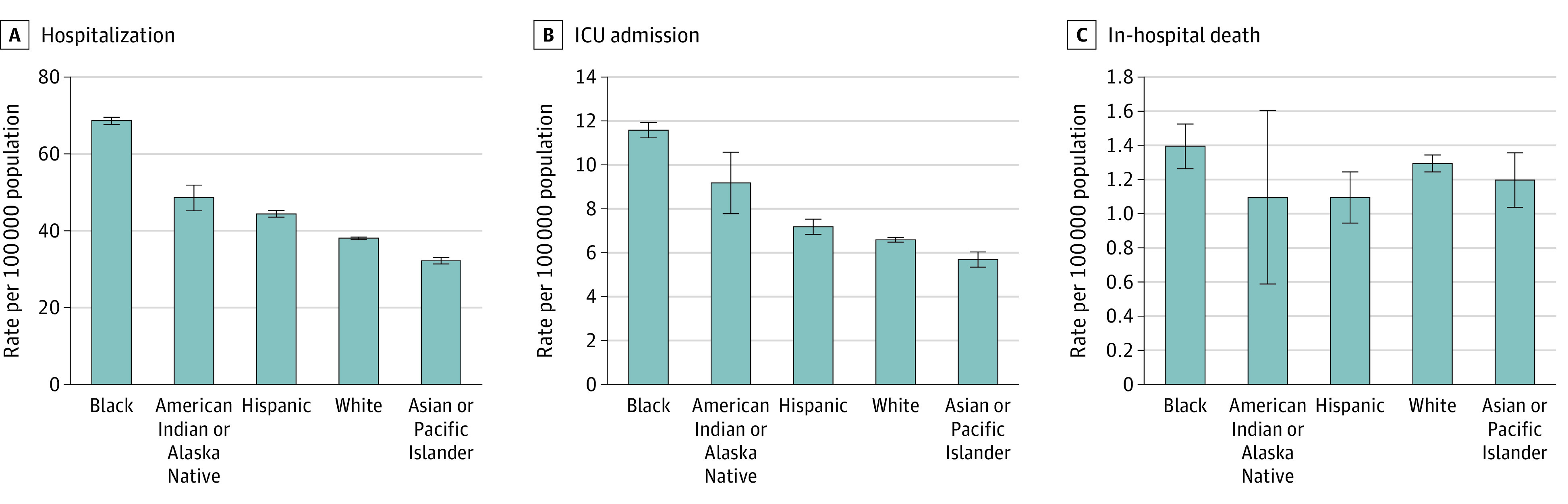
Overall Age-Adjusted Rates of Hospitalization, Intensive Care Unit (ICU) Admission, and In-Hospital Death by Race and Ethnicity

Age-adjusted rates per 100 000 population of hospitalization, ICU admission and in-hospital death are displayed in Figure 2 eTable 3 in the Supplement. Age-adjusted RRs for hospitalization and ICU admission are presented in Table 3. Age-adjusted hospitalization and ICU admission RRs were higher in Black persons compared with White persons across all seasons (Table 3). During the 2009 influenza A H1N1 pandemic season, age-adjusted hospitalization rates compared with White persons were higher among American Indian or Alaska Native persons (RR, 1.82; 95% CI, 1.48-2.22) and Hispanic persons (RR, 1.89; 95% CI, 1.77-2.02), and ICU admission rates were higher among Hispanic persons (RR, 1.77; 95% CI, 1.53-2.04). During that same season, rates of in-hospital death were higher among Hispanic persons (RR, 1.87; 95% CI, 1.28-2.74) compared with White persons (Table 3).

**Table 3.** Age-Adjusted Rate Ratios of Hospitalization, ICU Admission, and In-Hospital Death by Race and Ethnicity and Influenza Season

Outcome	Rate ratio (95% CI)										
	Overall	2009-2010	2010-2011	2011-2012	2012-2013	2013-2014	2014-2015	2015-2016	2016-2017	2017-2018	2018-2019
Hospitalization											
White	1 [Reference]	1 [Reference]	1 [Reference]	1 [Reference]	1 [Reference]	1 [Reference]	1 [Reference]	1 [Reference]	1 [Reference]	1 [Reference]	1 [Reference]
Black	1.81 (1.78-1.83)	2.17 (2.06-2.29)	2.10 (1.97-2.24)	1.82 (1.62-2.03)	1.63 (1.56-1.71)	1.86 (1.77-1.96)	1.58 (1.51-1.65)	1.99 (1.88-2.10)	1.73 (1.66-1.80)	1.65 (1.60-1.70)	1.72 (1.66-1.79)
American Indian or Alaska Native	1.28 (1.19-1.37)	1.82 (1.48-2.22)	1.16 (0.87-1.54)	2.82 (1.89-4.22)	1.03 (0.80-1.32)	1.67 (1.34-2.07)	0.95 (0.76-1.20)	1.63 (1.27-2.08)	1.15 (0.93-1.41)	0.90 (0.76-1.07)	1.72 (1.48-2.00)
Asian or Pacific Islander	0.85 (0.82-0.87)	0.95 (0.86-1.05)	0.83 (0.73-0.95)	1.46 (1.24-1.72)	0.72 (0.65-0.78)	0.63 (0.56-0.70)	0.79 (0.74-0.85)	0.82 (0.74-0.90)	0.94 (0.88-1.01)	0.80 (0.76-0.84)	0.68 (0.64-0.73)
Hispanic	1.17 (1.14-1.19)	1.89 (1.77-2.02)	1.41 (1.29-1.54)	1.62 (1.40-1.87)	1.10 (1.03-1.18)	1.29 (1.20-1.39)	0.91 (0.85-0.97)	1.40 (1.30-1.51)	0.98 (0.92-1.05)	0.92 (0.88-0.96)	1.22 (1.16-1.29)
ICU admission											
White	1 [Reference]	1 [Reference]	1 [Reference]	1 [Reference]	1 [Reference]	1 [Reference]	1 [Reference]	1 [Reference]	1 [Reference]	1 [Reference]	1 [Reference]
Black	1.75 (1.69-1.81)	1.94 (1.72-2.18)	1.68 (1.43-1.97)	1.47 (1.10-1.96)	1.59 (1.41-1.79)	1.53 (1.37-1.72)	1.62 (1.45-1.79)	1.77 (1.56-2.01)	1.98 (1.80-2.18)	1.71 (1.58-1.84)	1.59 (1.45-1.74)
American Indian or Alaska Native	1.39 (1.19-1.62)	1.54 (0.98-2.43)	1.03 (0.50-2.14)	3.12 (1.11-8.73)	0.81 (0.43-1.51)	1.45 (0.91-2.30)	1.02 (0.58-1.81)	1.38 (0.79-2.42)	1.45 (0.92-2.27)	1.17 (0.80-1.69)	2.17 (1.59-2.97)
Asian or Pacific Islander	0.87 (0.81-0.92)	0.95 (0.77-1.17)	0.97 (0.73-1.29)	1.19 (0.78-1.81)	0.72 (0.57-0.90)	0.42 (0.32-0.55)	0.91 (0.76-1.08)	0.82 (0.65-1.02)	1.08 (0.92-1.26)	0.88 (0.77-1.00)	0.71 (0.60-0.83)
Hispanic	1.09 (1.03-1.14)	1.77 (1.53-2.04)	1.29 (1.04-1.60)	1.26 (0.87-1.83)	0.94 (0.78-1.14)	0.94 (0.79-1.11)	0.87 (0.74-1.03)	1.33 (1.13-1.58)	0.92 (0.78-1.08)	0.93 (0.83-1.05)	1.10 (0.97-1.25)
In-hospital death											
White	1 [Reference]	1 [Reference]	1 [Reference]	1 [Reference]	1 [Reference]	1 [Reference]	1 [Reference]	1 [Reference]	1 [Reference]	1 [Reference]	1 [Reference]
Black	1.15 (1.04-1.27)	1.25 (0.87-1.79)	1.37 (0.90-2.09)	1.12 (0.50-2.51)	0.90 (0.61-1.32)	1.03 (0.74-1.42)	1.05 (0.80-1.38)	1.20 (0.81-1.76)	1.09 (0.84-1.43)	1.28 (1.05-1.56)	0.92 (0.69-1.23)
American Indian or Alaska Native	0.93 (0.59-1.44)	1.47 (0.47-4.61)	0	0	0.94 (0.23-3.80)	1.70 (0.63-4.58)	0.32 (0.04-2.26)	0	0.82 (0.20-3.39)	0.56 (0.18-1.78)	2.20 (0.96-5.02)
Asian or Pacific Islander	0.97 (0.84-1.11)	1.27 (0.79-2.06)	1.35 (0.76-2.40)	1.07 (0.33-3.45)	0.71 (0.41-1.24)	0.40 (0.21-0.78)	0.87 (0.59-1.27)	1.42 (0.90-2.24)	1.06 (0.75-1.49)	0.94 (0.71-1.25)	0.76 (0.52-1.13)
Hispanic	0.90 (0.79-1.04)	1.87 (1.28-2.74)	1.04 (0.57-1.88)	0.83 (0.28-2.50)	0.60 (0.33-1.08)	0.76 (0.47-1.24)	0.61 (0.39-0.96)	1.41 (0.90-2.19)	0.81 (0.54-1.20)	0.77 (0.56-1.05)	0.91 (0.64-1.29)

Abbreviation: ICU, intensive care unit.

Age-adjusted rates of hospitalization, ICU admission, and in-hospital death by race and ethnicity differed by surveillance site. While all sites experienced higher hospitalization and ICU admission rates in Black persons compared with White persons, only a few sites (ie, Colorado, Minnesota, and New Mexico) experienced higher hospitalization and ICU admission rates in American Indian or Alaska Native persons compared with White persons (eFigure 2 in the Supplement). Similarly, differences in rates of severe outcomes among Hispanic persons compared with White persons were most pronounced in certain sites (ie, Colorado, Connecticut, New Mexico, and Albany and Rochester, New York).

## Discussion

In this cross-sectional study using data from FluSurv-NET over the course of 10 influenza seasons, we identified age-stratum–specific racial and ethnic disparities in influenza-associated hospitalization, ICU admission, and in-hospital death rates, with the greatest disparities observed among younger age groups. Few rate differences were observed in adults aged 75 years or older across racial/ethnic groups. Among adults aged 18 to 64 years, hospitalization and ICU admission rates were higher for Black persons and American Indian or Alaska Native persons compared with White persons. Notably, differences in rates of hospitalization, ICU admission, and in-hospital deaths by race and ethnicity were greatest among children aged 4 years or younger. Overall, after adjusting for age, Black persons had the highest influenza-associated hospitalization rates, followed by American Indian or Alaska Native persons and Hispanic persons, with similar trends for ICU admission rates.

In our analysis, the most marked racial and ethnic disparities were observed for influenza-associated in-hospital deaths among children aged 4 years or younger. Black, Hispanic, and Asian or Pacific Islander children had 3- to 4-fold the rate of in-hospital death compared with White children. Consistent with our findings, reports using National Vital Statistics Surveillance mortality data have shown that disparities in pneumonia and influenza mortality are greatest in the youngest American Indian or Alaska Native children,^17^ and racial/ethnic disparities in infant and early life mortality have been identified for Black children and Hispanic children.^18,19^

Across all age groups, we found that Black persons had the highest rates of hospitalization and ICU admission, followed by American Indian or Alaska Native persons and Hispanic persons. Our findings are similar to prior studies that have identified disparities in influenza-associated hospitalizations in children during 2002 through 2009 using data from the New Vaccine Surveillance Network,^20^ and across all ages in 2 separate FluSurv-NET analyses.^12,13^ In one FluSurv-NET analysis that examined hospitalization rates by race and ethnicity and census-tract poverty level during 2010 to 2012,^12^ age-adjusted hospitalization rates were highest for Black persons; within each race and ethnicity group, hospitalization rates within high-poverty census tracts were nearly 2-fold the rates in low-poverty census tracts. Another analysis of FluSurv-NET data from 2009 to 2010 through 2013 to 2014 used a multilevel modeling approach to simultaneously assess individual-level and census tract–based determinants of influenza-associated hospitalization rates.^13^ In that analysis, individual factors associated with increased hospitalization rates included race (Black persons had 1.7-fold higher odds of hospitalization and Hispanic persons had 1.2-fold higher odds of hospitalization compared with White persons), and census tract–level factors included living in areas with higher levels of poverty and household crowding. However, outside of these earlier FluSurv-NET analyses and studies conducted during the 2009 influenza A H1N1 pandemic,^8,10,21^ data are relatively limited on the association of race and ethnicity with recent rates of severe influenza-associate disease. To our knowledge, this is the largest analysis to date to examine disparities in rates of multiple severe influenza-associated outcomes across all racial and ethnic groups.

In our analysis, while rates of in-hospital death were similar across racial and ethnic groups when combining seasons, during the 2009 influenza A H1N1 pandemic season, rates of in-hospital mortality were highest in Hispanic persons, followed by American Indian or Alaska Native persons. Our findings are consistent with a 2009 H1N1 pandemic study by Dee et al,^9^ which found that the proportion of H1N1pdm09-associated pediatric deaths was disproportionately higher among Hispanic children compared with the distribution of Hispanic children in the US population. A study by Quinn et al^21^ found significant racial/ethnic disparities in exposure risk, susceptibility to complications, and access to care during the 2009 pandemic. Generally, it has been recognized that a pandemic influenza outbreak in the United States would likely disproportionately impact socially disadvantaged groups, and pandemic planning must consider exposure, susceptibility, and treatment of high-risk populations.^8^

Striking racial and ethnic disparities in COVID-19–associated hospitalization rates have been observed during the ongoing COVID-19 pandemic, with differences observed between non-Hispanic White persons and other racial and ethnic groups being far more pronounced than for both seasonal and 2009 H1N1 pandemic influenza. Compared with rates among White persons, cumulative COVID-19–associated hospitalization rates are approximately 3-fold higher in Hispanic persons aged 17 years or younger, 6-fold higher in American Indian or Alaska Native persons aged 18 to 49 years, 4-fold higher among American Indian or Alaska Native persons aged 50 to 64 years, and 2-fold higher among Black persons 65 years or older.^22^ Multiple studies have shown that racial and ethnic disparities associated with COVID-19 are likely due to inequities in social determinants of health among racial and ethnic minority groups. Contributing factors include lack of access to quality health care, disproportionate representation in essential occupations, and crowded living conditions, which may all increase exposure to COVID-19.^5,6,7,23,24,25,26^ Future work should investigate whether racial and ethnic disparities in influenza-associated hospitalizations and outcomes are influenced by similar factors.

Seasonal influenza vaccination coverage disparities by race and ethnicity have been well-described,^27,28,29,30^ which might partially explain why racial and ethnic minority groups, such as Black, Hispanic, Asian or Pacific Islander, and American Indian or Alaska Native populations, are disproportionately affected by severe influenza and also helps to identify critical gaps in influenza vaccination that need to be filled. Over multiple influenza seasons through 2015, vaccination coverage in the general population has been lower among Black and Hispanic adults compared with White adults, and Black and Hispanic children had lower rates of full vaccination coverage compared with White children^27,28,29,30^; our findings in hospitalized patients of all ages showed similar disparities. Vaccination coverage has been shown to correlate with socioeconomic status,^31^ access to health care, and birth country, where those with greater access to care had higher vaccination coverage, and those born outside the United States had lower vaccination coverage compared with US-born persons.^29^

### Limitations

This study has several limitations. First, FluSurv-NET conducts surveillance in selected counties within the United States, and findings may not represent the entire US population. Second, laboratory-confirmed influenza-associated hospitalizations reported to FluSurv-NET are identified by clinician-directed testing. Thus, hospitalization rates may be underestimated, since they have not been adjusted for testing practices, which are known to differ by surveillance site, age group, and timing during influenza seasons,^32^ and may also vary by race and ethnicity. Third, approximately 16% of persons were missing ethnicity and were classified based only on their reported race. Fourth, death rates reflect persons who died during their hospitalization, and our analysis did not capture influenza-associated deaths that occurred after hospital discharge. Preliminary analyses from FluSurv-NET indicate that deaths occurring within 30 days after discharge could account for a substantial burden of seasonal influenza-associated mortality, particularly among older adults.^33^ Fifth, because these data were not available for the underlying source population, this analysis did not adjust rates for other factors, such as vaccination status, underlying medical conditions, receipt of antivirals, or illness severity at hospital admission, all of which could potentially impact findings.

## Conclusions

This cross-sectional study identified racial and ethnic disparities in rates of influenza-associated hospitalization, ICU admission, and in-hospital death from the 2009 to 2010 through 2018 to 2019 influenza seasons, with the widest disparities occurring among young children from racial/ethnic minority groups, such as Black, Hispanic, and Asian or Pacific Islander children. These data could help target prevention and intervention efforts, such as improved influenza vaccine coverage and early use of antiviral treatment, to improve influenza-associated outcomes among racial and ethnic minority groups.

## Notes

Supplement. **eFigure 1. **FluSurv-NET Surveillance Catchment Areas, 2018 to 2019 **eTable 1.** Clinical Characteristics of Persons With Laboratory-Confirmed Influenza-Associated Hospitalization by Race and Ethnicity **eTable 2.** Age-Specific Rates of Hospitalization, ICU Admission and In-Hospital Death by Race/Ethnicity **eTable 3.** Age-Adjusted Rates of Hospitalization, ICU Admission, and In-Hospital Death by Race/Ethnicity and Season **eFigure 2.** Age-Adjusted Rates of Hospitalization, ICU Admission, and In-Hospital Death by Surveillance Site

## References

[1] Centers for Disease Control and Prevention. Disease burden of influenza. Accessed June 9, 2021. https://www.cdc.gov/flu/about/burden/index.html

[2] Ingraham NE, Purcell LN, Karam BS, Racial/ethnic disparities in hospital admissions from COVID-19 and determining the impact of neighborhood deprivation and primary language. medRxiv. Preprint posted online September 22, 2020. doi:10.1101/2020.09.02.20185983 PMC8130213 34003427

[3] Moore JT, Ricaldi JN, Rose CE, ; COVID-19 State, Tribal, Local, and Territorial Response Team. Disparities in incidence of COVID-19 among underrepresented racial/ethnic groups in counties identified as hotspots during June 5-18, 2020—22 states, February-June 2020. MMWR Morb Mortal Wkly Rep. 2020;69(33):1122-1126. doi:10.15585/mmwr.mm6933e1 32817602 PMC7439982

[4] Muñoz-Price LS, Nattinger AB, Rivera F, . Racial disparities in incidence and outcomes among patients with COVID-19. JAMA Netw Open. 2020;3(9):e2021892. doi:10.1001/jamanetworkopen.2020.21892 32975575 PMC7519420

[5] Ko JY, Danielson ML, Town M, ; COVID-NET Surveillance Team. Risk factors for coronavirus disease 2019 (COVID-19)-associated hospitalization: COVID-19-Associated Hospitalization Surveillance Network and Behavioral Risk Factor Surveillance System. Clin Infect Dis. 2021;72(11):e695-e703. doi:10.1093/cid/ciaa1419 32945846 PMC7543371

[6] Killerby ME, Link-Gelles R, Haight SC, ; CDC COVID-19 Response Clinical Team. Characteristics associated with hospitalization among patients with COVID-19—Metropolitan Atlanta, Georgia, March-April 2020. MMWR Morb Mortal Wkly Rep. 2020;69(25):790-794. doi:10.15585/mmwr.mm6925e1 32584797 PMC7316317

[7] Rossen LM, Branum AM, Ahmad FB, Sutton P, Anderson RN. Excess deaths associated with COVID-19, by age and race and ethnicity—United States, January 26-October 3, 2020. MMWR Morb Mortal Wkly Rep. 2020;69(42):1522-1527. doi:10.15585/mmwr.mm6942e2 33090978 PMC7583499

[8] Blumenshine P, Reingold A, Egerter S, Mockenhaupt R, Braveman P, Marks J. Pandemic influenza planning in the United States from a health disparities perspective. Emerg Infect Dis. 2008;14(5):709-715. doi:10.3201/eid1405.071301 18439350 PMC2600245

[9] Dee DL, Bensyl DM, Gindler J, . Racial and ethnic disparities in hospitalizations and deaths associated with 2009 pandemic influenza A (H1N1) virus infections in the United States. Ann Epidemiol. 2011;21(8):623-630. doi:10.1016/j.annepidem.2011.03.002 21737049

[10] Centers for Disease Control and Prevention. Deaths related to 2009 pandemic influenza A (H1N1) among American Indian/Alaska Natives—12 states, 2009. MMWR Morb Mortal Wkly Rep. 2009;58(48):1341-1344. 20010508

[11] Grohskopf LA, Alyanak E, Broder KR, . Prevention and control of seasonal influenza with vaccines: recommendations of the Advisory Committee on Immunization Practices—United States, 2020-21 influenza season. MMWR Recomm Rep. 2020;69(8):1-24. doi:10.15585/mmwr.rr6908a1 32820746 PMC7439976

[12] Hadler JL, Yousey-Hindes K, Pérez A, . Influenza-related hospitalizations and poverty levels—United States, 2010-2012. MMWR Morb Mortal Wkly Rep. 2016;65(5):101-105. doi:10.15585/mmwr.mm6505a1 26866729

[13] Chandrasekhar R, Sloan C, Mitchel E, . Social determinants of influenza hospitalization in the United States. Influenza Other Respir Viruses. 2017;11(6):479-488. doi:10.1111/irv.12483 28872776 PMC5720587

[14] Chaves SS, Lynfield R, Lindegren ML, Bresee J, Finelli L. The US Influenza Hospitalization Surveillance Network. Emerg Infect Dis. 2015;21(9):1543-1550. doi:10.3201/eid2109.141912 26291121 PMC4550140

[15] Chow EJ, Rolfes MA, O’Halloran A, . Acute cardiovascular events associated with influenza in hospitalized adults : a cross-sectional study. Ann Intern Med. 2020;173(8):605-613. doi:10.7326/M20-1509 32833488 PMC8097760

[16] Centers for Disease Control and Prevention. U.S. census populations with bridged race categories. Accessed June 9, 2021. https://www.cdc.gov/nchs/nvss/bridged_race.htm

[17] Groom AV, Hennessy TW, Singleton RJ, Butler JC, Holve S, Cheek JE. Pneumonia and influenza mortality among American Indian and Alaska Native people, 1990-2009. Am J Public Health. 2014;104(suppl 3):S460-S469. doi:10.2105/AJPH.2013.301740 24754620 PMC4035860

[18] Rogers RG, Lawrence EM, Hummer RA, Tilstra AM. Racial/ethnic differences in early-life mortality in the United States. Biodemography Soc Biol. 2017;63(3):189-205. doi:10.1080/19485565.2017.1281100 29035105 PMC5729754

[19] Ely DM, Driscoll AK. Infant mortality in the United States, 2018: data from the period linked birth/infant death file. Natl Vital Stat Rep. 2020;69(7):1-18. 32730740

[20] Iwane MK, Chaves SS, Szilagyi PG, . Disparities between black and white children in hospitalizations associated with acute respiratory illness and laboratory-confirmed influenza and respiratory syncytial virus in 3 US counties—2002-2009. Am J Epidemiol. 2013;177(7):656-665. doi:10.1093/aje/kws299 23436899

[21] Quinn SC, Kumar S, Freimuth VS, Musa D, Casteneda-Angarita N, Kidwell K. Racial disparities in exposure, susceptibility, and access to health care in the US H1N1 influenza pandemic. Am J Public Health. 2011;101(2):285-293. doi:10.2105/AJPH.2009.188029 21164098 PMC3020202

[22] Centers for Disease Control and Prevention. COVID-19 racial and ethnic health disparities. Accessed June 10, 2021. https://www.cdc.gov/coronavirus/2019-ncov/community/health-equity/racial-ethnic-disparities/disparities-hospitalization.html

[23] Price-Haywood EG, Burton J, Fort D, Seoane L. Hospitalization and mortality among Black patients and White patients with COVID-19. N Engl J Med. 2020;382(26):2534-2543. doi:10.1056/NEJMsa2011686 32459916 PMC7269015

[24] Selden TM, Berdahl TA. COVID-19 And racial/ethnic disparities in health risk, employment, and household composition. Health Aff (Millwood). 2020;39(9):1624-1632. doi:10.1377/hlthaff.2020.00897 32663045

[25] Gold JAW, Wong KK, Szablewski CM, . Characteristics and clinical outcomes of adult patients hospitalized with COVID-19—Georgia, March 2020. MMWR Morb Mortal Wkly Rep. 2020;69(18):545-550. doi:10.15585/mmwr.mm6918e1 32379729 PMC7737948

[26] Rogers TN, Rogers CR, VanSant-Webb E, Gu LY, Yan B, Qeadan F. Racial disparities in COVID-19 mortality among essential workers in the United States. World Med Health Policy. 2020. doi:10.1002/wmh3.358 32837779 PMC7436547

[27] Lu PJ, O’Halloran A, Bryan L, . Trends in racial/ethnic disparities in influenza vaccination coverage among adults during the 2007-08 through 2011-12 seasons. Am J Infect Control. 2014;42(7):763-769. doi:10.1016/j.ajic.2014.03.021 24799120 PMC5822446

[28] Lu PJ, O’Halloran A, Williams WW, Lindley MC, Farrall S, Bridges CB. Racial and ethnic disparities in vaccination coverage among adult populations in the U.S. Vaccine. 2015;33(suppl 4):D83-D91. doi:10.1016/j.vaccine.2015.09.031 26615174

[29] Williams WW, Lu PJ, O’Halloran A, . Surveillance of vaccination coverage among adult populations—United States, 2015. MMWR Surveill Summ. 2017;66(11):1-28. doi:10.15585/mmwr.ss6611a1 28472027 PMC5829683

[30] Santibanez TA, Grohskopf LA, Zhai Y, Kahn KE. Complete influenza vaccination trends for children six to twenty-three months. Pediatrics. 2016;137(3):e20153280. doi:10.1542/peds.2015-3280 26908692 PMC5751428

[31] de Figueiredo A, Johnston IG, Smith DM, Agarwal S, Larson HJ, Jones NS. Forecasted trends in vaccination coverage and correlations with socioeconomic factors: a global time-series analysis over 30 years. Lancet Glob Health. 2016;4(10):e726-e735. doi:10.1016/S2214-109X(16)30167-X 27569362

[32] Reed C, Chaves SS, Daily Kirley P, . Estimating influenza disease burden from population-based surveillance data in the United States. PLoS One. 2015;10(3):e0118369. doi:10.1371/journal.pone.0118369 25738736 PMC4349859

[33] McGowan C, Arriola C, Cummings C, . Causes of in-hospital and post discharge mortality among patients hospitalized with laboratory-confirmed influenza, Influenza Hospitalization Surveillance Network, 2014–2015. Open Forum Infect Dis. 2017;4(suppl 1):S24. doi:10.1093/ofid/ofx162.061

