# Supplementary material for: Rates of Influenza-Associated Hospitalization, Intensive Care Unit Admission, and In-Hospital Death by Race and Ethnicity in the United States From 2009 to 2019
Source: JAMA Netw Open. 2021 Aug 24;4(8):e2121880. doi: 10.1001/jamanetworkopen.2021.21880 (PMC8385599; doi:10.1001/jamanetworkopen.2021.21880)
Supplement: Supplement. — eFigure 1. FluSurv-NET Surveillance Catchment Areas, 2018 to 2019 eTable 1. Clinical Characteristics of Persons With Laboratory-Confirmed Influenza-Associated Hospitalization by Race and Ethnicity eTable 2. Age-Specific Rates of Hospitalization, ICU Admission and In-Hospital Death by Race/Ethnicity eTable 3. Age-Adjusted Rates of Hospitalization, ICU Admission, and In-Hospital Death by Race/Ethnicity and Season eFigure 2. Age-Adjusted Rates of Hospitalization, ICU Admission, and In-Hospital Death by Surveillance Site [file jamanetwopen-e2121880-s001.pdf]

Supplemental Online Content

O'Halloran AC, Holstein R, Cummings C, et al. Rates of influenza-associated hospitalization, intensive care unit admission, and in-hospital death by race and ethnicity in the United States from 2009 to 2019. *JAMA Netw Open*. 2021;4(8):e2121880. doi:10.1001/jamanetworkopen.2021.21880

eFigure 1. FluSurv-NET Surveillance Catchment Areas, 2018 to 2019

eTable 1. Clinical Characteristics of Persons With Laboratory-Confirmed Influenza-Associated Hospitalization by Race and Ethnicity

eTable 2. Age-Specific Rates of Hospitalization, ICU Admission and In-Hospital Death by Race/Ethnicity

eTable 3. Age-Adjusted Rates of Hospitalization, ICU Admission, and In-Hospital Death by Race/Ethnicity and Season

eFigure 2. Age-Adjusted Rates of Hospitalization, ICU Admission, and In-Hospital Death by Surveillance Site

This supplemental material has been provided by the authors to give readers additional information about their work.

© 2021 O'Halloran AC et al. *JAMA Network Open.*

eFigure 1. FluSurv-NET Surveillance Catchment Areas, 2018 to 2019

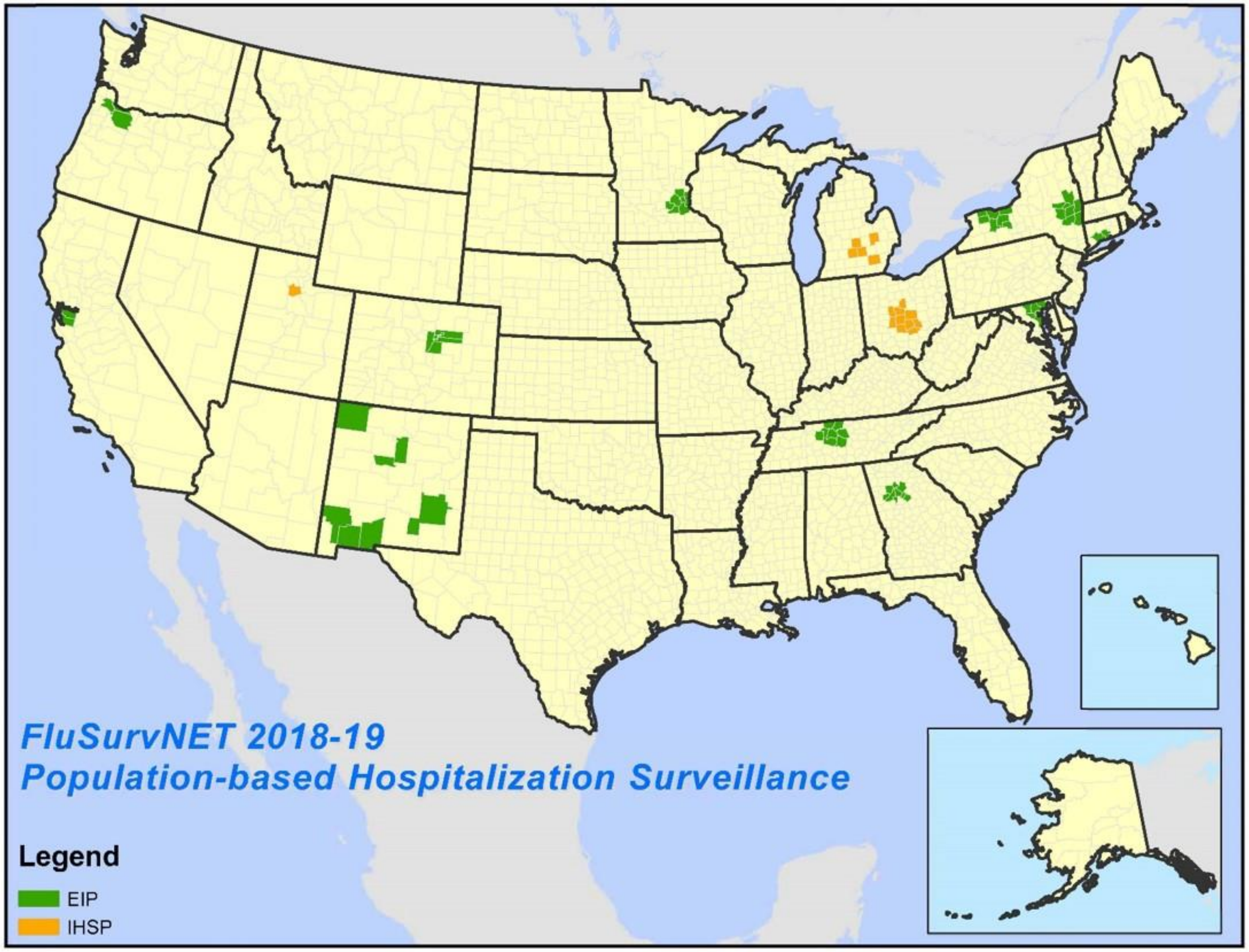

Abbreviations: EIP = Emerging Infections Program surveillance sites; IHSP = Influenza Hospitalization Surveillance Project surveillance sites

This map displays counties included in FluSurv-NET surveillance during the 2018-19 influenza season with exceptions for prior seasons as noted: **California** (Alameda, Contra Costa, and San Francisco counties); **Colorado** (Adams, Arapahoe, Denver, Douglas, and Jefferson counties);

© 2021 O'Halloran AC et al. *JAMA Network Open.*

Connecticut (New Haven, Middlesex (2010-11 through 2018-19),Hartford (2010-11 through 2011-12); **Georgia** (Clayton, Cobb, DeKalb, Douglas, Fulton, Gwinnett, Newton, and Rockdale counties); **Maryland** (Anne Arundel, Baltimore, Baltimore City, Carroll, Harford, Howard); **Michigan** (Clinton, Eaton, Genesee (2012-13 through 2018-19), Ingham, and Washtenaw (2017-18 through 2018-19); **Minnesota** (Anoka, Carver, Dakota, Hennepin, Ramsey, Scott, and Washington counties); **New Mexico** (Bernalillo, Chaves, Dona Ana, Grant, Luna, San Juan (2010-11 through 2018-19), and Santa Fe counties); **New York** (Albany, Columbia, Genesee, Greene, Livingston, Monroe, Montgomery, Ontario, Orleans, Rensselaer, Saratoga, Schenectady, Schoharie, Wayne, and Yates counties); **Ohio** (Delaware, Fairfield, Franklin, Licking, Madison, Morrow, Pickaway and Union (2010-11 through 2018-19), Hocking and Perry (2014-15 through 2018-19)); **Oregon** (Clackamas, Multnomah, and Washington counties); **Tennessee** (Cheatham, Davidson, Dickson, Robertson, Rutherford, Sumner, Williamson, and Wilson counties); **Utah** (Salt Lake County (2010-11 through 2018-19)); **Iowa** (Bremer, Buena Vista, Cherokee, Decatur, Delaware, Des Moines, Emmet, Fayette, Floyd, Greene, Grundy, Hamilton, Hancock, Humboldt, Jasper, Johnson, Jones, Marshall, Monroe, O'Brien, Polk, Pottawattamie, Poweshiek, Scott counties included in 2009-10; Dallas, Guthrie, Madison, Polk, Warren counties included in 2012-13); **Idaho** (Ada, Bingham, Kootenai counties included in 2009-10 through 2010-11); **North Dakota (**Williams county included in 2009-10); **Oklahoma** (Cherokee, Comanche, Garfield, Oklahoma, Pontotoc counties included in 2009-10 through 2010-11); **Rhode Island** (Providence county included in 2010-11 through 2012-13; **South Dakota**: Brown, Lincoln, Minnehaha, Pennington counties included in 2009-10)

© 2021 O'Halloran AC et al. *JAMA Network Open.*

eTable 1. Clinical Characteristics of Persons With Laboratory-Confirmed Influenza-Associated Hospitalization by Race and Ethnicity

	NH White Unweighted N (Weighted %)	NH Black Unweighted N (Weighted %)	Hispanic Unweighted N (Weighted %)	NH A/PI Unweighted N (Weighted %)	NH AI/AN Unweighted N (Weighted %)
0-4 years					
**Underlying Medical Conditions**[a]					
Asthma	374 (12.7)	569 (24.1)	296 (12.7)	74 (13.4)	20 (13.6)
Chronic lung disease	146 (4.9)	181 (7.7)	98 (4.2)	32 (5.8)	14 (9.5)
Cardiovascular disease	186 (6.3)	192 (8.1)	150 (6.4)	43 (7.8)	14 (9.5)
Chronic metabolic disease	55 (1.9)	60 (2.5)	39 (1.7)	8 (1.4)	6 (4.1)
Renal Disease	45 (1.5)	26 (1.1)	27 (1.2)	2 (0.4)	7 (4.8)
Neurologic/Neuromuscular	288 (9.7)	289 (12.2)	204 (8.8)	58 (10.5)	13 (8.8)
Immunocompromised	143 (4.8)	99 (4.2)	75 (3.2)	19 (3.4)	4 (2.7)
Blood disorders	38 (1.3)	278 (11.8)	23 (1.0)	12 (2.2)	2 (1.4)
Liver disease[b]	8 (0.4)	9 (0.5)	5 (0.3)	1 (0.2)	1 (0.9)
Obesity[c]	131 (4.7)	99 (4.6)	86 (4.0)	27 (5.2)	11 (7.6)
**Vaccination**[d]	923 (41.1)	595 (31.8)	639 (37.2)	193 (42.8)	54 (45.4)
**Antiviral Treatment**	2318 (75.1)	1882 (76.4)	1800 (75.9)	463 (77.3)	127 (82.5)
5-17 years					
**Underlying Medical Conditions**[a]					
Asthma	872 (32.2)	1067 (55.9)	496 (38.9)	103 (31.6)	20 (31.7)
Chronic lung disease	206 (7.6)	108 (5.7)	83 (6.5)	22 (6.7)	2 (3.2)
Cardiovascular disease	198 (7.3)	132 (6.9)	75 (5.9)	23 (7.1)	3 (4.8)
Chronic metabolic disease	234 (8.6)	87 (4.6)	99 (7.8)	20 (6.1)	5 (7.9)
Renal Disease	88 (3.2)	43 (2.3)	50 (3.9)	12 (3.7)	2 (3.2)
Neurologic/Neuromuscular	563 (20.8)	283 (14.8)	257 (20.2)	69 (21.2)	11 (17.5)
Immunocompromised	337 (12.4)	178 (9.3)	159 (12.5)	48 (14.7)	4 (6.3)
Blood disorders	78 (2.9)	472 (24.7)	40 (3.1)	16 (4.9)	0 (.)
Liver disease[b]	9 (0.5)	12 (0.9)	12 (1.3)	5 (2.1)	0 (.)
Obesity[c]	349 (13.0)	306 (16.8)	269 (22.0)	31 (10.4)	19 (29.7)
**Vaccination**[d]	987 (35.6)	567 (29.1)	397 (30.9)	117 (35.5)	21 (32.8)
**Antiviral Treatment**	2080 (75.3)	1544 (79.5)	1024 (79.8)	263 (79.9)	53 (82.8)

© 2021 O'Halloran AC et al. *JAMA Network Open.*

18-49 years					
**Underlying Medical Conditions**[a]					
Asthma	2908 (29.1)	2661 (38.2)	900 (27.6)	189 (20.2)	79 (34.5)
Chronic lung disease	1343 (13.4)	621 (8.9)	200 (6.1)	44 (4.7)	17 (7.4)
Cardiovascular disease	1388 (13.9)	1296 (18.6)	314 (9.6)	98 (10.5)	35 (15.3)
Chronic metabolic disease	2420 (24.2)	1575 (22.6)	724 (22.2)	208 (22.3)	71 (31.0)
Renal Disease	630 (6.3)	915 (13.1)	251 (7.7)	95 (10.2)	30 (13.1)
Neurologic/Neuromuscular	1616 (16.2)	849 (12.2)	310 (9.5)	94 (10.1)	22 (9.6)
Immunocompromised	1585 (15.9)	1455 (20.9)	406 (12.4)	114 (12.2)	25 (10.9)
Blood disorders	347 (3.5)	542 (7.8)	69 (2.1)	22 (2.4)	6 (2.6)
Liver disease[b]	401 (5.0)	162 (2.9)	113 (4.5)	36 (4.8)	23 (13.1)
Obesity[c]	3858 (38.9)	2926 (43.0)	1183 (37.7)	248 (27.9)	109 (47.6)
**Vaccination**[d]	2821 (27.8)	1573 (22.4)	739 (22.6)	243 (25.6)	62 (26.4)
**Antiviral Treatment**	8629 (85.6)	5951 (85.0)	2845 (86.9)	837 (88.7)	200 (85.8)
50-64 years					
**Underlying Medical Conditions**[a]					
Asthma	3083 (21.8)	2073 (30.1)	521 (26.1)	190 (23.4)	59 (27.4)
Chronic lung disease	5646 (39.7)	2316 (33.9)	405 (20.1)	140 (17.2)	55 (26.0)
Cardiovascular disease	5323 (37.4)	2871 (42.1)	599 (29.6)	308 (38.3)	72 (33.9)
Chronic metabolic disease	5743 (40.2)	3032 (44.0)	1045 (52.0)	437 (53.0)	117 (55.8)
Renal Disease	1828 (12.7)	1558 (23.0)	341 (17.3)	200 (24.7)	49 (23.8)
Neurologic/Neuromuscular	2542 (17.8)	1082 (15.8)	218 (10.8)	77 (9.9)	33 (15.3)
Immunocompromised	3010 (21.1)	1716 (25.2)	382 (19.0)	151 (18.4)	41 (19.0)
Blood disorders	585 (4.0)	284 (4.0)	61 (2.9)	22 (2.6)	4 (1.9)
Liver disease[b]	985 (7.8)	644 (10.5)	188 (10.6)	42 (6.5)	25 (14.8)
Obesity[c]	6705 (47.0)	3364 (49.5)	927 (46.5)	226 (27.8)	98 (46.5)
**Vaccination**[d]	5736 (39.9)	2309 (33.3)	673 (33.7)	301 (37.0)	87 (41.2)
**Antiviral Treatment**	12360 (86.6)	5850 (86.4)	1748 (87.3)	742 (89.7)	191 (89.7)

© 2021 O'Halloran AC et al. *JAMA Network Open.*

65-74 years					
**Underlying Medical Conditions**[a]					
Asthma	1975 (16.7)	737 (23.7)	288 (24.0)	141 (18.5)	8 (12.7)
Chronic lung disease	5173 (43.2)	1142 (36.2)	355 (29.7)	162 (20.3)	20 (21.3)
Cardiovascular disease	6587 (55.5)	1766 (57.0)	530 (45.6)	344 (46.1)	35 (46.8)
Chronic metabolic disease	5737 (48.3)	1725 (55.9)	714 (61.0)	424 (56.2)	45 (56.6)
Renal Disease	2274 (19.0)	945 (31.0)	281 (25.1)	217 (29.5)	16 (20.4)
Neurologic/Neuromuscular	2472 (20.4)	637 (20.6)	172 (15.6)	112 (15.4)	13 (18.1)
Immunocompromised	2611 (22.1)	646 (20.0)	193 (16.5)	126 (15.4)	14 (18.0)
Blood disorders	501 (3.8)	146 (4.2)	30 (2.8)	18 (2.3)	4 (4.3)
Liver disease[b]	442 (4.2)	224 (8.1)	59 (5.8)	35 (5.0)	3 (6.5)
Obesity[c]	5201 (44.0)	1354 (43.9)	503 (43.1)	140 (19.1)	32 (44.4)
**Vaccination**[d]	6587 (54.8)	1330 (42.8)	570 (49.4)	392 (51.5)	42 (54.7)
**Antiviral Treatment**	10457 (88.3)	2663 (88.3)	1023 (88.7)	694 (91.5)	70 (88.7)
75+ years					
**Underlying Medical Conditions**[a]					
Asthma	2818 (11.0)	490 (16.2)	267 (16.6)	318 (17.4)	10 (9.6)
Chronic lung disease	8079 (30.8)	869 (26.6)	440 (28.6)	458 (24.9)	30 (30.1)
Cardiovascular disease	18038 (68.8)	2017 (62.5)	917 (57.4)	1080 (58.4)	68 (66.6)
Chronic metabolic disease	12107 (46.1)	1655 (52.5)	913 (57.0)	914 (49.5)	59 (58.0)
Renal Disease	6594 (25.4)	1132 (36.5)	404 (25.6)	588 (32.8)	30 (27.7)
Neurologic/Neuromuscular[b]	8702 (32.8)	1112 (34.9)	458 (28.9)	533 (28.4)	27 (30.8)
Immunocompromised	3526 (13.2)	431 (13.7)	190 (12.3)	175 (9.3)	8 (9.5)
Blood disorders	1110 (3.9)	127 (3.6)	48 (2.6)	38 (1.7)	3 (2.4)
Liver disease[c]	291 (1.2)	64 (2.2)	37 (2.9)	43 (2.3)	0 (.)
Obesity[d]	6354 (24.4)	939 (29.6)	419 (26.1)	200 (10.7)	27 (25.7)
**Vaccination**[e]	16567 (62.6)	1449 (46.0)	840 (52.1)	1032 (56.0)	65 (62.8)
**Antiviral Treatment**	23501 (89.8)	2797 (89.1)	1456 (91.2)	1705 (92.6)	91 (90.6)

Abbreviations: NH = Non-Hispanic; AI/AN= American Indian/Alaska Native; Hispanic = Hispanic or Latino; A/PI = Asian/Pacific Islander

[a] Underlying medical conditions are not mutually exclusive

[b] Neurologic disease not collected until the 2011-12 season

[c] Liver disease not collected until the 2011-12 season

[d] Obesity not collected until Sept 1, 2009 of the 2009-10 season

© 2021 O'Halloran AC et al. *JAMA Network Open.*

[e] Receipt of at least one dose of current season influenza vaccine among persons ≥6 months. Up to 4 sources (medical chart, immunization, registry, primary care provider, and patient interview) were used to ascertain vaccination status.

© 2021 O'Halloran AC et al. *JAMA Network Open.*

eTable 2. Age-Specific Rates of Hospitalization, ICU Admission and In-Hospital Death by Race/Ethnicity

	NH White	NH Black	NH AI/AN	Hispanic	NH A/PI
	Rate[a] (95% CI)	Rate[a] (95% CI)	Rate[a] (95% CI)	Rate[a] (95% CI)	Rate[a] (95% CI)
**Hospitalization**					
0-4 years	33.6 (32.4, 34.8)	74.4 (71.4, 77.3)	100.9 (85.0, 116.9)	62.8 (60.3, 65.4)	42.4 (39.0, 45.8)
5-17 years	11.1 (10.7, 11.5)	22.1 (21.1, 23.1)	16.5 (12.5, 20.5)	14.2 (13.4, 15)	9.0 (8.0, 10.0)
18-49 years	13.6 (13.3, 13.9)	34.2 (33.4, 35.0)	23.4 (20.4, 26.4)	17.5 (16.9, 18.1)	8.3 (7.7, 8.8)
50-64 years	39.8 (39.2, 40.4)	99.4 (97.1, 101.6)	61.1 (53.0, 69.3)	49.8 (47.7, 51.9)	24.9 (23.3, 26.5)
65-74 years	90.5 (89.0, 92.0)	157.8 (152.6, 162.9)	86.9 (69.8, 104.0)	107.2 (101.5, 112.9)	75.7 (71.0, 80.5)
75+ years	257.2 (254.3, 260.1)	270.5 (261.9, 279.2)	202.9 (167.0, 238.7)	240.4 (229.8, 250.9)	261.5 (251.2, 271.9)
**ICU Admission**					
0-4 years	5.8 (5.3-6.3)	15.9 (14.5-17.2)	20.3 (13.2-27.5)	11.3 (10.3-12.4)	7.6 (6.1-9.0)
5-17 years	2.5 (2.3-2.7)	4.9 (4.5-5.4)	4.6 (2.5-6.8)	2.9 (2.5-3.2)	2.4 (1.9-2.9)
18-49 years	2.8 (2.7-2.9)	5.2 (4.9-5.5)	5.2 (3.8-6.6)	3.2 (2.9-3.5)	1.6 (1.4-1.8)
50-64 years	8.4 (8.2-8.7)	17.7 (16.7-18.7)	9.9 (6.6-13.1)	8.8 (7.9-9.6)	5.1 (4.4-5.9)
65-74 years	17.0 (16.3-17.6)	25.5 (23.4-27.5)	22.8 (14.0-31.6)	18.9 (16.5-21.3)	14.7 (12.6-16.8)
75+ years	32.2 (31.2-33.2)	40.4 (37.1-43.8)	23.1 (11.0-35.2)	28.2 (24.6-31.8)	38.9 (34.9-42.9)

© 2021 O'Halloran AC et al. *JAMA Network Open.*

In-Hospital Death					
0-4 years	0.1 (0.0-0.2)	0.3 (0.1-0.5)	0.7 (0.0-1.9)	0.3 (0.1-0.5)	0.4 (0.1-0.8)
5-17 years	0.1 (0.1-0.2)	0.1 (0.1-0.2)	0.5 (0.0-1.2)	0.1 (0.0-0.2)	0.2 (0.0-0.3)
18-49 years	0.3 (0.3-0.4)	0.4 (0.3-0.5)	0.7 (0.2-1.2)	0.3 (0.3-0.4)	0.2 (0.1-0.3)
50-64 years	1.4 (1.2-1.5)	2.1 (1.8-2.4)	1.7 (0.3-3.0)	1.5 (1.1-1.8)	0.6 (0.4-0.9)
65-74 years	2.9 (2.6-3.2)	3.5 (2.7-4.2)	1.8 (0.0-4.2)	3.1 (2.1-4.1)	2.9 (2.0-3.8)
75+ years	11.4 (10.8-12.0)	10.6 (8.9-12.3)	5.0 (0.0-10.6)	8.1 (6.2-10.1)	13.8 (11.5-16.2)

Abbreviations: CI = Confidence Interval; NH = Non-Hispanic; AI/AN= American Indian/Alaska Native; Hispanic = Hispanic or Latino; A/PI = Asian/Pacific Islander; ICU = Intensive Care Unit

[a]Rate per 100,000 population

© 2021 O'Halloran AC et al. *JAMA Network Open.*

eTable 3. Age-Adjusted Rates of Hospitalization, ICU Admission, and In-Hospital Death by Race/Ethnicity and Season

	All seasons	2009-10	2010-11	2011-12	2012-13	2013-14	2014-15	2015-16	2016-17	2017-18	2018-19
	Rate[a] (95% CI)	Rate[a] (95% CI)	Rate[a] (95% CI)	Rate[a] (95% CI)	Rate[a] (95% CI)	Rate[a] (95% CI)	Rate[a] (95% CI)	Rate[a] (95% CI)	Rate[a] (95% CI)	Rate[a] (95% CI)	Rate[a] (95% CI)
**Hospitalization**											
NH White	38.1 (37.9-38.4)	24.2 (23.5-25.0)	16.4 (15.9-17.0)	6.3 (6.0-6.7)	37.3 (36.5-38.2)	28.8 (28.0-29.5)	52.1 (51.1-53.1)	24.2 (23.5-24.9)	50.5 (49.5-51.5)	89.1 (87.8-90.4)	53.6 (52.6-54.6)
NH Black	68.8 (68.0-69.7)	52.6 (50.3-54.9)	34.6 (32.7-36.5)	11.5 (10.4-12.6)	61.0 (58.4-63.5)	53.5 (51.1-55.8)	82.2 (79.1-85.2)	48.1 (45.9-50.3)	87.2 (84.2-90.2)	147.1 (143.2-151.0)	92.4 (89.4-95.4)
NH AI/AN	48.7 (45.4-52.0)	44.0 (35.2-52.8)	19.0 (13.5-24.4)	17.8 (10.8-24.9)	38.4 (28.8-48.0)	47.9 (37.6-58.3)	49.6 (38.3-61.0)	39.5 (29.9-49.1)	58.0 (46.0-70.0)	80.7 (67.0-94.3)	92.3 (78.4-106.2)
Hispanic	44.5 (43.6-45.4)	45.9 (43.2-48.5)	23.2 (21.3-25.1)	10.2 (8.9-11.6)	41.1 (38.4-43.9)	37.2 (34.7-39.7)	47.2 (44.2-50.2)	33.8 (31.5-36.2)	49.7 (46.7-52.7)	82.0 (78.2-85.7)	65.4 (62.3-68.6)
NH A/PI	32.3 (31.4-33.1)	23.1 (20.8-25.3)	13.7 (12.0-15.4)	9.2 (7.8-10.7)	26.7 (24.4-29.1)	18.1 (16.2-20.0)	41.3 (38.4-44.2)	19.8 (17.9-21.7)	47.8 (44.7-50.8)	71.4 (67.9-75.0)	36.7 (34.1-39.2)
**ICU Admission**											
NH White	6.6 (6.5-6.7)	5.4 (5.1-5.8)	3.1 (2.9-3.4)	1.1 (1.0-1.3)	6.0 (5.7-6.4)	7.0 (6.6-7.4)	7.9 (7.5-8.3)	4.9 (4.5-5.2)	7.8 (7.4-8.2)	13.5 (13.0-14.0)	9.7 (9.3-10.1)
NH Black	11.6 (11.2-11.9)	10.6 (9.5-11.6)	5.3 (4.5-6.0)	1.7 (1.2-2.1)	9.6 (8.6-10.6)	10.7 (9.7-11.8)	12.8 (11.6-14.0)	8.6 (7.7-9.5)	15.5 (14.2-16.8)	23.1 (21.5-24.6)	15.4 (14.2-16.6)
NH AI/AN	9.2 (7.8-10.6)	8.4 (4.6-12.2)	3.2 (0.9-5.6)	3.6 (0.0-7.2)	4.9 (1.8-7.9)	10.1 (5.5-14.8)	8.1 (3.5-12.7)	6.7 (3.0-10.4)	11.3 (6.3-16.4)	15.7 (9.9-21.6)	21.1 (14.6-27.6)
Hispanic	7.2 (6.9-7.6)	9.6 (8.4-10.9)	4.1 (3.3-4.9)	1.4 (0.9-1.9)	5.7 (4.7-6.7)	6.5 (5.5-7.6)	6.9 (5.8-8.0)	6.5 (5.5-7.5)	7.2 (6.1-8.3)	12.6 (11.2-14.0)	10.7 (9.5-12.0)
NH A/PI	5.7 (5.4-6.1)	5.2 (4.1-6.2)	3.0 (2.2-3.9)	1.4 (0.8-1.9)	4.3 (3.4-5.3)	2.9 (2.2-3.7)	7.2 (6.0-8.4)	4.0 (3.1-4.8)	8.5 (7.2-9.7)	11.9 (10.4-13.3)	6.8 (5.8-7.9)
**Death**											
NH White	1.3 (1.2-1.3)	0.9 (0.7-1.0)	0.6 (0.5-0.7)	0.2 (0.1-0.2)	1.1 (1.0-1.2)	1.2 (1.1-1.4)	1.8 (1.6-1.9)	0.8 (0.6-0.9)	1.7 (1.6-1.9)	2.7 (2.5-2.9)	1.6 (1.5-1.8)
NH Black	1.4 (1.3-1.6)	1.1 (0.7-1.4)	0.8 (0.5-1.1)	0.2 (0.1-0.4)	1.0 (0.6-1.3)	1.3 (0.9-1.6)	1.9 (1.4-2.3)	0.9 (0.6-1.2)	1.9 (1.4-2.4)	3.5 (2.8-4.1)	1.5 (1.1-1.9)
NH AI/AN	1.2 (0.6-1.7)	1.3 (0.0-2.7)	0.0	0.0	1.0 (0.0-2.5)	2.1 (0.0-4.1)	0.6 (0.0-1.7)	0.0	1.4 (0.0-3.4)	1.5 (0.0-3.3)	3.6 (0.6-6.5)
Hispanic	1.1 (1.0-1.3)	1.6 (1.1-2.2)	0.6 (0.3-0.9)	0.2 (0.0-0.3)	0.7 (0.3-1.0)	0.9 (0.5-1.4)	1.1 (0.6-1.5)	1.1 (0.6-1.5)	1.4 (0.9-1.9)	2.1 (1.5-2.7)	1.5 (1.0-2.0)
NH A/PI	1.2 (1.1-1.4)	1.1 (0.6-1.6)	0.8 (0.4-1.2)	0.2 (0.0-0.4)	0.8 (0.4-1.2)	0.5 (0.2-0.8)	1.5 (1.0-2.1)	1.1 (0.6-1.5)	1.8 (1.2-2.4)	2.6 (1.9-3.2)	1.2 (0.8-1.7)

Abbreviations: NH = Non-Hispanic; AI/AN= American Indian/Alaska Native; Hispanic = Hispanic or Latino; A/PI = Asian/Pacific Islander; Ref = Reference group; ICU = Intensive Care Unit

[a]Rate per 100,000 population

© 2021 O'Halloran AC et al. *JAMA Network Open.*

eFigure 2. Age-Adjusted Rates of Hospitalization, ICU Admission, and In-Hospital Death by Surveillance Site

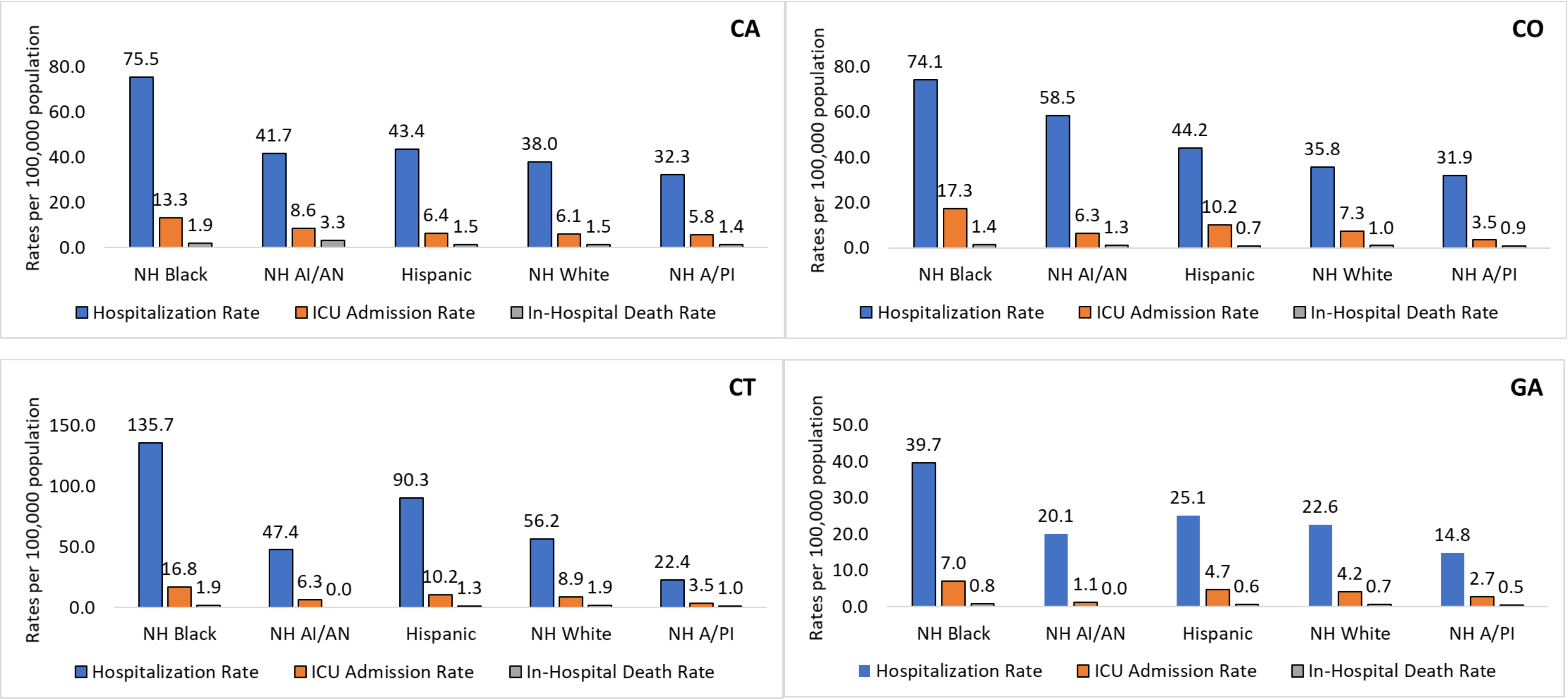

© 2021 O’Halloran AC et al. *JAMA Network Open.*

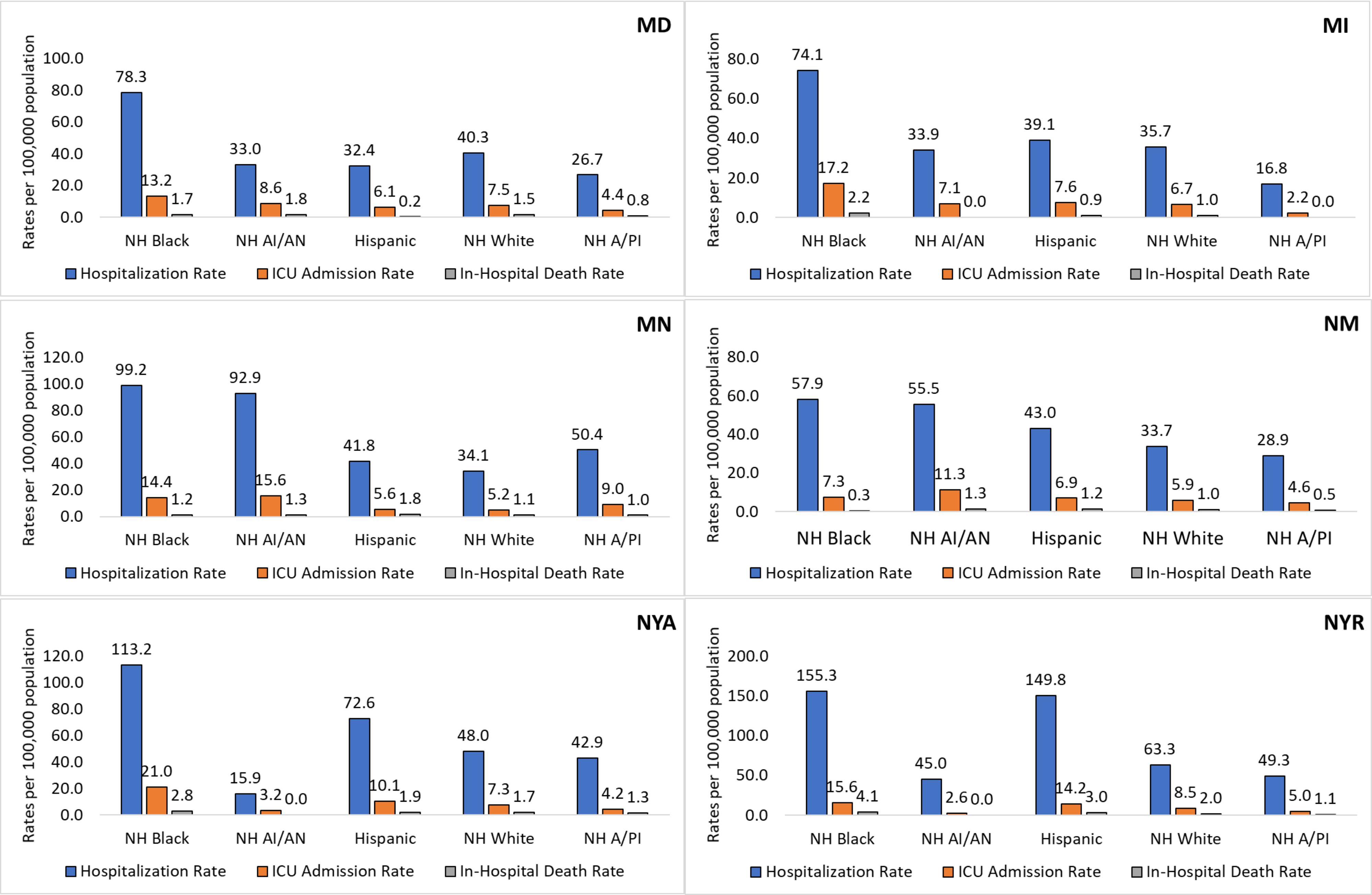

© 2021 O'Halloran AC et al. *JAMA Network Open.*

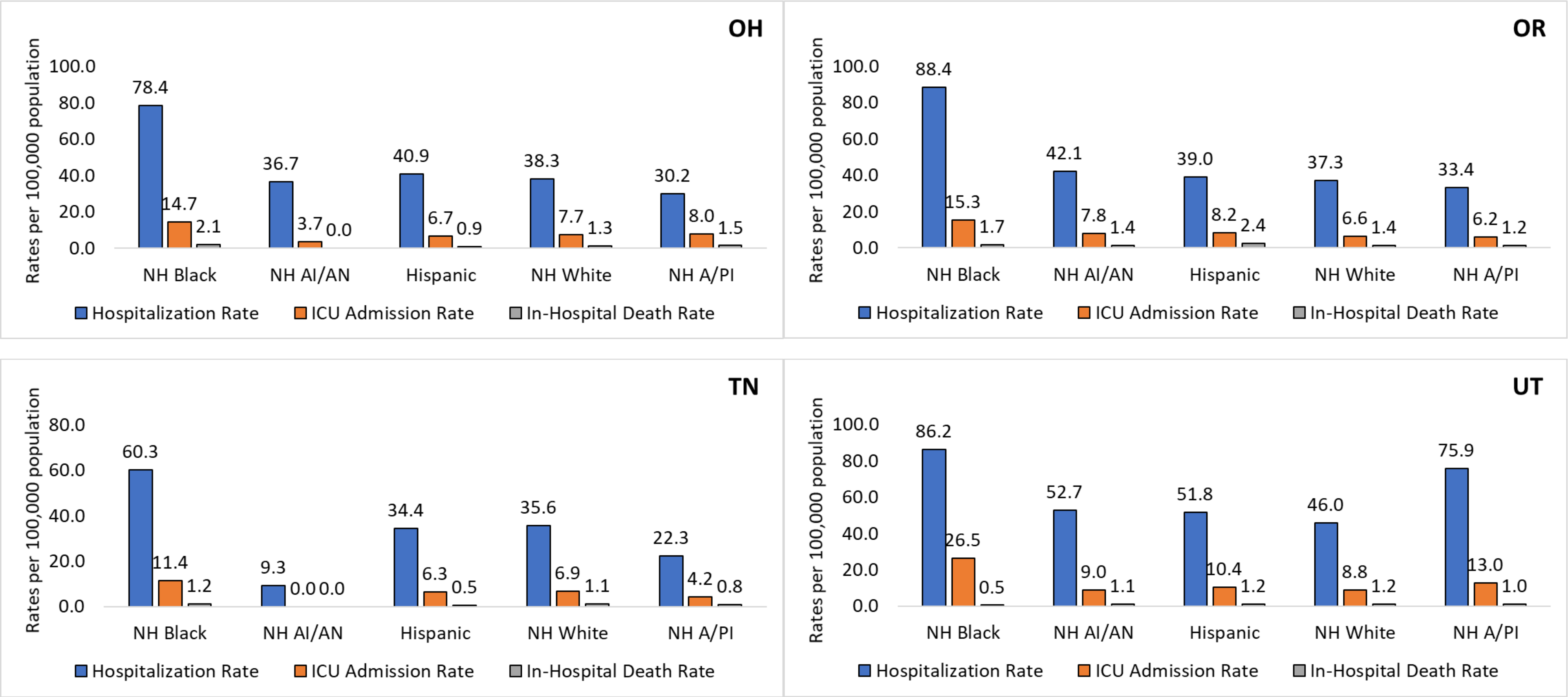

Abbreviations: NH = Non-Hispanic; AI/AN= American Indian/Alaska Native; Hispanic = Hispanic or Latino; A/PI = Asian/Pacific Islander; Ref = Reference group; ICU = Intensive Care Unit; NYA = New York Albany; NYR = New York Rochester

[a] Site-level data not shown for 6 sites who participated in FluSurv-NET surveillance for ≤3 of the 10 influenza seasons (IA, ID, ND, OK, RI, SD).

© 2021 O'Halloran AC et al. *JAMA Network Open.*
